# Surveillance of Dengue Fever Virus: A Review of Epidemiological Models and Early Warning Systems

**DOI:** 10.1371/journal.pntd.0001648

**Published:** 2012-05-22

**Authors:** Vanessa Racloz, Rebecca Ramsey, Shilu Tong, Wenbiao Hu

**Affiliations:** 1 School of Population Health, University of Queensland, Brisbane, Queensland, Australia; 2 School of Public Health and Institute of Health and Biomedical Innovation, Queensland University of Technology, Kelvin Grove Campus, Kelvin Grove, Queensland, Australia; NASA Goddard Space Flight Center, United States of America

## Abstract

Dengue fever affects over a 100 million people annually hence is one of the world's most important vector-borne diseases. The transmission area of this disease continues to expand due to many direct and indirect factors linked to urban sprawl, increased travel and global warming. Current preventative measures include mosquito control programs, yet due to the complex nature of the disease and the increased importation risk along with the lack of efficient prophylactic measures, successful disease control and elimination is not realistic in the foreseeable future. Epidemiological models attempt to predict future outbreaks using information on the risk factors of the disease. Through a systematic literature review, this paper aims at analyzing the different modeling methods and their outputs in terms of acting as an early warning system. We found that many previous studies have not sufficiently accounted for the spatio-temporal features of the disease in the modeling process. Yet with advances in technology, the ability to incorporate such information as well as the socio-environmental aspect allowed for its use as an early warning system, albeit limited geographically to a local scale.

## Author Summary

Despite mass vaccination campaigns and large scaled improvements in global surveillance, infectious diseases are a worldwide problem. In recent years, the ability to use models as a tool to help visualize, understand and combat infectious diseases has become more feasible and reliable. In this context, modelling focuses on transmission patterns between the different animal, human or vector components as well as including parameters which affect these pathways such as environmental, climatic or geographic ones. The output of these models can help in decision making processes concerning control purposes, surveillance methods and hopefully also as good predictive tools. Prediction forms part of surveillance systems, and more specifically in early warning systems. It is the timely collection and analysis of data as well as the use of risk-based assessments in order to aid in prompt health interventions such as movement control, vaccination campaigns or the distribution of important information. Early warning systems for vector borne diseases are especially complex due to the involvement of various factors originating from the human, animal and insect sector as well the disease itself. The authors investigate the variety and depth of available models for dengue fever surveillance and their use as early warning tools.

## Introduction

### Dengue fever virus

Dengue fever (DF) is one of the most common widespread vector borne diseases in the world [1], [2], [3], [4]. There are currently 2.5 billion people living in areas at risk of DF transmission, with 100 million cases reported annually [5], [6]. DF is a flaviviral disease caused by one of four serotypes of dengue virus (DEN 1–4) which are transmitted by mosquito vectors, in particular the peridomestic species *Aedes aegypti* [2], [7], and *Ae. albopictus*, which has recently been expanding its geographic distribution as seen in several outbreaks [8].

Infection by one serotype will provide lifelong immunity to that particular strain but not to the remaining three [1], [9]. Cross-strain infections are common and can have severe consequences, with extreme cases leading to death [10]. Over the past 40 years the incidence and geographic distribution of DF has increased in many countries, particularly in those with tropical and sub-tropical climates [6], [11], [12], [13], [14]. DF has strong spatial and temporal patterns which have been linked to climatic and environmental conditions [15]. Thus the inclusion of spatial and temporal data in analytic processes may potentially allow for the identification of DF characteristics linked to these parameters and have significant applications in the prevention and control of this disease. Additionally, as discussed in the Intergovernmental Panel on Climate Change report [16], with global temperatures likely to increase, it is predicted that the endemic range of DF will expand geographically [17], [18], [19], [20], [21]. Altered extrinsic incubations periods (EIP), biting rates hence transmission levels [18], [22] of the disease will increase its capacity as a vector, more specifically its competence and activity, and is linked to climate and environment, amongst other factors [23].

### Surveillance of vector borne diseases

Several surveillance system methods exist for a variety of vector borne diseases [24], [25], yet successful early warning strategies are limited due to the complex and dynamic nature of the disease, environmental factors, the vectors and the hosts involved as well as the necessary health system infrastructure needed to combine all the factors in an integrated manner. In Europe, the VBORNET network which combines knowledge from entomologists and public health experts [26] was recently developed with aim at building an integrated approach to surveillance of vector borne diseases. The report highlights the different parameters and methods needed to establish surveillance activities, as well as the various data types and collection strategies (www.vbornet.eu).

Sentinel surveillance is a type of risk based surveillance which can serve as an early warning system, and has had some general success in terms of prediction for diseases such as Bluetongue disease [27], Rift Valley fever [28] and West Nile [29]. The main objective of an early warning system is the collection of information leading to timely decision making processes which trigger disease intervention strategies in order to reduce the burden and effect of the disease on a specified population.

As summarised in Beatty et al., 2010, recommendations suggest a comprehensive approach to dengue fever virus control, with emphasis on mosquito control, environmental measures, efficient data collection and sharing platforms including laboratory networks and finally the development of an early detection system [30].

Yet the use of actual epidemiological models for early warning predictions in vector borne diseases is more constraint. In recent years, mapping methods have been tried in terms of forecasting risk zones for vector borne diseases as described in Bergquist 2011, through the use of satellite based data [31]. Recent developments studying the combination of mapping and mathematical modelling will be discussed further.

As with many infectious diseases, one of the success measures of a surveillance system depends on the ability to predict an imminent outbreak through an early warning system. The process of identifying a potential threat and targeting surveillance and control methods form part of an early warning system. Such an approach is categorized as a targeted surveillance system as opposed to random surveillance [32]. This is an important difference in order to increase the probability of detection of any first or repeated incursion of disease at the earliest time possible. The ability to create an early warning system through the combination of climate, environmental, host and vector based data through various processes such as mathematical modelling and Geographical Information System (GIS) mapping have been used in many ways to improve veterinary and public health surveillance systems [33]. The combination of different prediction, surveillance and control methods and the tools involved in each process present a great potential in the combat against a variety of disease as described in Eisen & Eisen 2011 [34]. This paper aims at providing an insight into the current DF surveillance and modelling processes and the implementation of their outputs in terms of applicability as an early warning system.

## Methods

**Figure 1:**
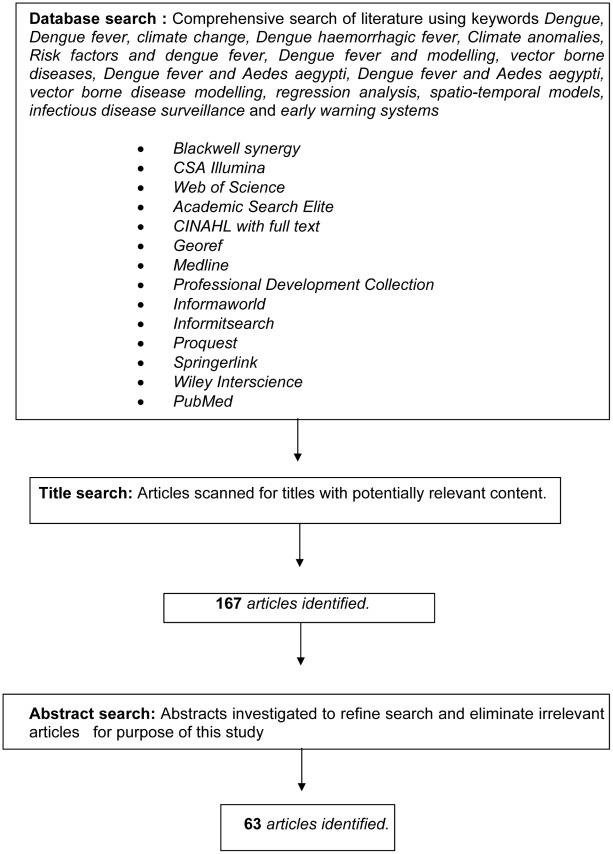
Graphical summary of the literature search process.

Through a comprehensive literature review, major databases including Blackwell synergy, CSA Illumina, Web of Science, Academic Search Elite, CINAHL with full text, Georef, medline, Professional Development Collection, Informaworld, InformitSearch, Proquest, Springerlink, Wiley Interscience and Pubmed (http://www.ncbi.nlm.nih.gov/pubmed) were searched. The key words used in this literature search were *Dengue*, *Dengue fever*, *climate change*, *Dengue haemorrhagic fever*, *Climate anomalies*, *Risk factors and dengue fever*, *Dengue fever and modelling*, *vector borne diseases*, *Dengue fever and Aedes aegypti*, *Dengue fever and Aedes aegypti*, *vector borne disease modelling*, *regression analysis*, *spatio-temporal models*, *infectious disease surveillance* and *early warning systems*. Studies were included if the use of one or more epidemiological models were reported. During the initial search, studies were selected based on a review of titles and abstracts. Full studies were retrieved and reviewed for all relevant articles as seen in Figure 1.

**Figure 2:**
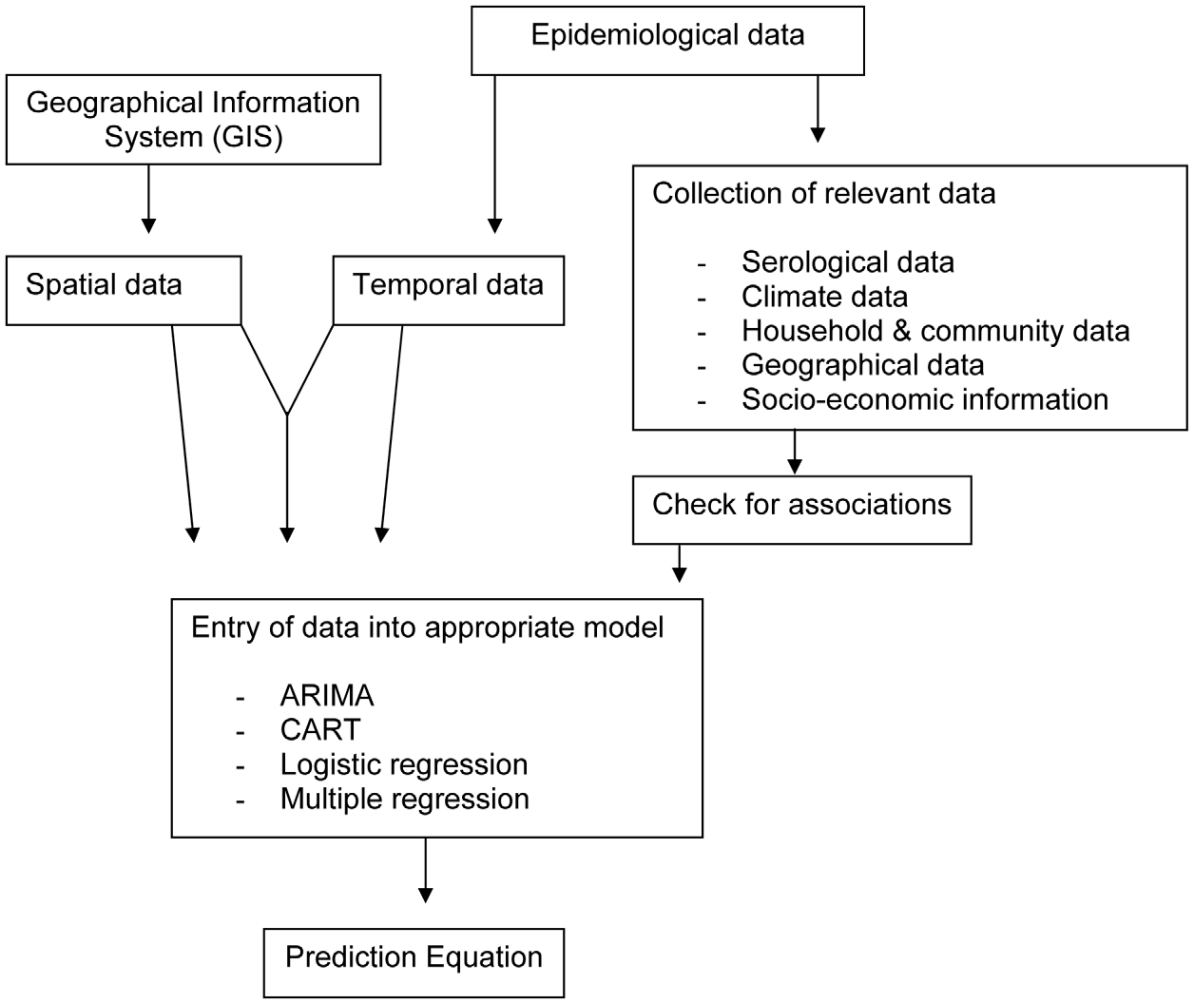
Flow chart process for data incorporation in dengue fever outbreak modelling.

**Figure 3:**
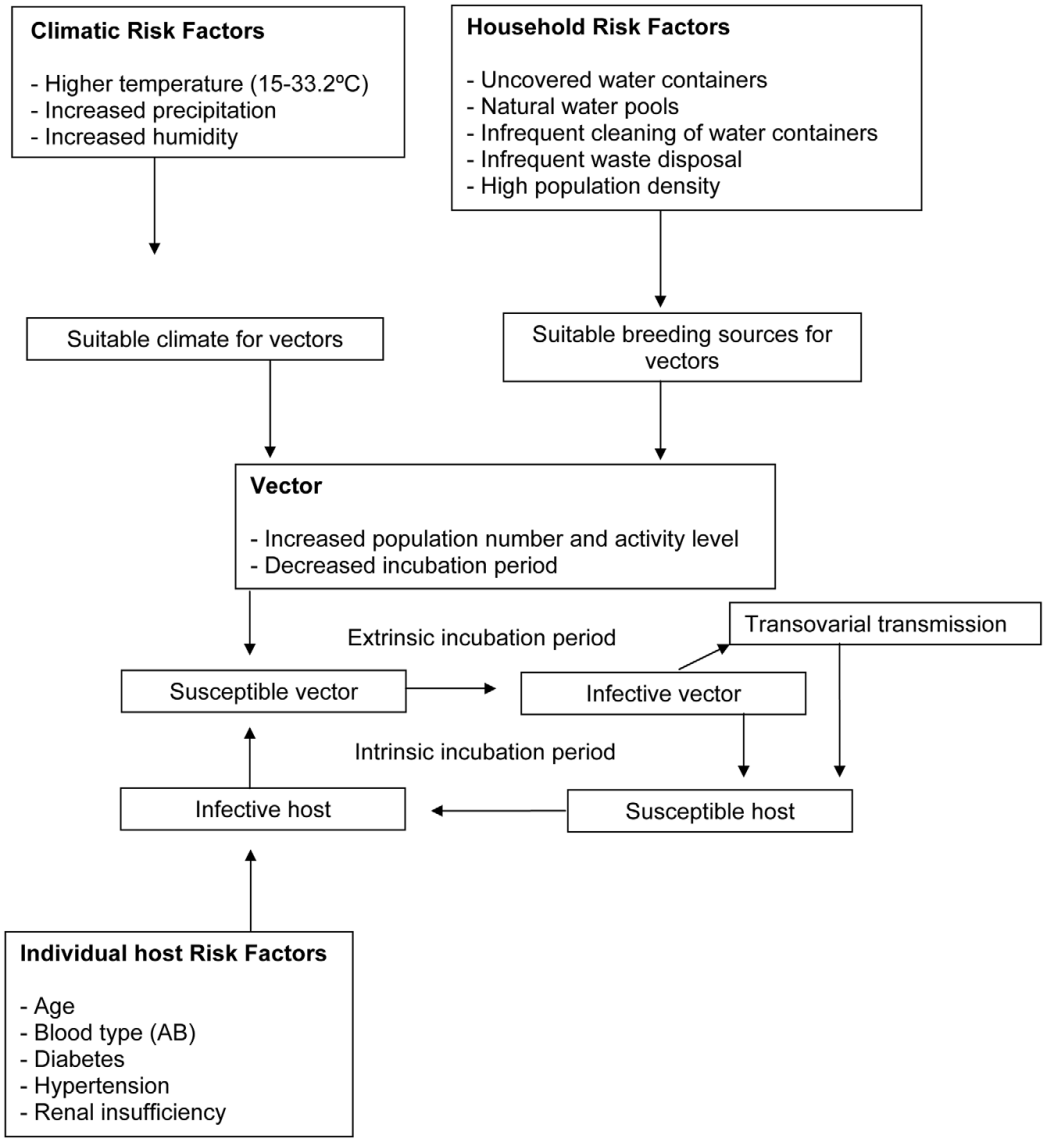
Transmission pathway and risk factors involved in dengue fever outbreaks.

In order to analyse the DF models, it was important to review the background information as well as the method used in output generation (Figure 2). Due to the difference in output objectives, biological factors, spatio-temporal parameters, geographical scales and mathematical equations used in more current models, the comparison of efficacy between models is complex. A synopsis of the different pathways and risk factors found in the literature review is shown in Figure 3.

## Results

Although many studies use a combination of epidemiological tools, three main focus areas were identified: 1) mapping tools, 2) mathematical models and finally 3) a combination of 1 and 2. The purpose of the maps and models are aimed at dengue reporting and surveillance, usually based on risk factors although recent studies have been introducing disease forecasting as their main objective.

As seen in Table 1, different categories for the analysis of the models existed, such as spatial scale, data collection time frame, model type and finally the incorporated risk factors. Although the main countries in the study were Argentina, Australia, Brazil, China, Cuba, India, Indonesia, Mexico, Puerto Rico, Singapore, Thailand and the USA, the actual spatial scale used in the models varied from community level to multi-country. Collection time points spanned from daily measures to biannual analysis. Although the mathematical basis of many of the models shared a common regression point, these varied from logistic, autoregressive, spatio-temporal or Poisson equations. Finally, one of the most encompassing and diverse parameters were the risk factors used in the dengue model creation such as temperature, precipitation, vegetation indices, wind velocity or even hygienic markers.

**Table 1:** Setting and parameters used in predictive dengue model creation.

Spatial scale	Collection time frame	Model	Risk factors
Community	Daily	Poisson	Temperature
Parish	Weekly	Time-series	Precipitation
District	Monthly	Autoregressive [75]	Wind velocity
Municipality	Bi-monthly	Multiple regression	Sea surface temperature
Province	Annually	Step-wise regression	Humidity
City	Bi-annually	Logistic regression	Geographical settings
State		Autoregressive Integrated Moving Average (ARIMA)	Hygienic parameters
Country		Classification & Regression Tree (CART)	Socio environmental factors
Multi- country		Spatio-temporal regression	Proximity to potential artificial breeding sights
			Vegetation dynamics

There were a number of different models capable of producing prediction equations for the transmission of dengue fever. The type of model selected was dependent on the type of data collected and the nature of the variables (Figure 2), and due to the subtle differences involved in each outbreak, no universal models existed for analysis and prediction.

Traditionally, the data usually consisted of serologic and environmental or socioeconomic variables. Recently, socio-environmental changes have been identified as important determinants in the transmission of DF, and spatial and temporal aspects of these changes have been increasingly incorporated into studies [35]. The inclusion of spatial data allows for the identification of spatial patterns of occurrence and the ability to identify areas at high risk of disease. The majority of previous studies in the past decade have implemented logistic or multiple regression models to identify possible risk factors. A drawback of these models is that they are not capable of accounting for autocorrelation in time-series data, which may limit the predictive capabilities of the resultant model.

### Mapping tools in dengue surveillance

In the past decade, mapping techniques and software have been able to incorporate a range of variables according to available data including socio-demographic, ecological, disease attributed factors, household infestation levels as well as many other climate, host or vector based ones [36] [34]. These maps allow for the risk visualisation of the disease through different avenues, be it human and/or vector associated.

Mapping tools for dengue surveillance also exist for more specific aspects on human derived risk factors [37], land based factors [38] or from a vector point of view [39]. The information on these more focussed maps is more detailed than for maps which combine many factors together yet might not allow for large scaled conclusions to be made concerning surveillance recommendations, giving each level of mapping tool its advantages and disadvantages.

### Mathematical methods in dengue surveillance

Similarly with the mapping techniques, mathematical models exist for a whole range of factors affecting dengue virus disease transmission. Mathematical models for dengue fever range from simple compartmental SEIR transmission equations, to complex equations involving the dynamics between human (DENSiM) and mosquito (CIMSiM) population dynamics and dengue transmission [40], [41]. The ability to combine various parameters adds complexity to sensitivity analyses [42], yet due to the intricate nature of vector borne diseases is a necessary measure. Recent models focus on climate driven factors such as correlating dengue cases to temperature or relative humidity, and as discussed further on, sea surface temperature and proximity to water bodies has also been analysed.

In terms of choice of mathematical methods, the AutoRegressive Integrated Moving Average (ARIMA) and Seasonal AutoRegressive Integrated Moving Average (SARIMA) models, which have the ability to cope with stochastic dependence of consecutive data, have become well established in the commercial and industrial fields [43], [44]. A DF study in Queensland, Australia used ARIMA modelling to examine the relationship between weather variables and the disease [45]. The implementation of SARIMA accounts for auto-correlations in time-series as well as seasonality, long-term trends and lags. Consequently, SARIMA has higher predictive capabilities than other models described above. However, this approach requires the input of a large amount of data meaning that SARIMA may not be suitable for studies with a small sample sizes. SARIMA is also based on the assumption of normality. For diseases that are rare or occur less frequently, the assumption of normality may not be met and thus SARIMA might not be an appropriate choice.

### Combined GIS and mathematical models for dengue surveillance

Combining GIS and mathematical models also exist for a range of dengue related parameters as seen in a study by Khormi & Kumar, 2011 [46], whereby socioeconomic parameters were used to show the relationship between dengue cases and spatial data in Saudi Arabia through Geographically Weighted Regression (GWR) analysis. Another example studies the relationship between population density and water supply in Vietnam through GIS and the Ross-MacDonald mathematical model using the basic reproductive number (R_0_) [47].

Another approach to predicting the spatial dynamics of both human dengue cases in relation to vector presence was presented through ecological niche modelling using GARP (Genetic Algorithm for Rule-set Prediction) in combination with GIS ecological landscape maps of Mexico [48]. The model allowed for an average predictive value of 80% in terms of forecasting mosquito occurrence in Mexico, a useful component in dengue surveillance. The concept of ecological niche (similar to climatic suitability envelope) modelling, defines how key climatic, environmental and topographical variables form a niche which is occupied by a specific species [27].

The depth and scope reached by combining maps and mathematical models provide a realistic platform to base surveillance and control decisions, as well as aiding in predicting outbreaks, yet limitations still occur in spatial and temporal terms. This is not including the extra risk factors which are difficult to forecast such as the introduction of dengue fever virus through infected human or mosquito into a potential hot spot.

### Early warning systems in dengue

As seen in Table 1, Figures 2 and 3, there are several different data collection and analysis pathways used to model DF transmission and intervention strategies. In terms of using the outputs of these models, two main objectives were identified: the use of DF models as a retrospective and validating method, and as an early warning tool to predict potential epidemics. Retrospective models use data as a validation method as seen in [49] for DF in Peru where data from 1994 to 2006 was analysed, and validated in latter epidemics throughout the region by evaluating the degree of association with demographic and geographic variables. Such techniques also allow for intervention and control strategies to be tested firstly on a hypothetical level, and then applied in the field, as seen in Luz et al [50]. In this study, epidemiological and economic assessments of different vector control strategies were tested in the city of Rio de Janeiro in Brazil in relation to DF.

Although many models discussed above include risk factors involving basic climate and household information, the calculations are mainly based on human and vector borne parameters. Through the advancement and access to technology, various software programs and improvements in database infrastructure allow for the use of multidimensional values to be included in models in order to progress from a purely applied mathematically based theme to more a dynamic one. Especially important in developing countries, is the role of resource-limited settings in the development of timely prediction tools. Chang et al [51], demonstrate how the use of Google Earth and GIS mapping technologies can aid in dengue surveillance, especially where unplanned urbanisation, a risk factor for the disease, is abundant. Another advantage of using web based tools including Google search functions is demonstrated in Chan et al [52], whereby dengue fever case reporting was collected in a quicker time frame than normally available in traditional official sources, and easily applied into a mapping visualization tool.

A large expanse of surveillance methods involved in dengue as well as the various combinations of parameters used for epidemiological modelling were found in the literature. As mentioned, the models include different transmission mechanisms, clinical manifestation data, current disease and vector control methods, treatment options, socio economic and risk factors of DF as well as the potential of developing into dengue hemorrhagic fever. The risk factors for developing DF included biological, human, vector, environmental, socio-demographic data as well as climate and parameters linked to climate change (Figure 3).

The ability to combine the GIS techniques with statistical and mathematical models with the intended output being a spatio-temporal tool for early warning system is not impossible given the quality and availability of data from surveillance systems as well as advanced technologies. As noted by LaDeau et al [53], due to the primary host of dengue being humans, not only must an early warning system be based on vector presence and activity but also on the complex nature of human movement and organisation. The role of imported cases can serve as a basic type of dengue outbreak early warning based on human movement [54], especially when certain climatic conditions are suitable for disease spread into local areas. Although this method is spatially limited, it is an important indicator considering the amount of travel which exists into dengue endemic areas.

## Discussion

As seen in the reports and studies reviewed, there are a large number of environmentally related as well as disease based parameters which influence the intensity, frequency, location and spread of a DF outbreak. Several limitations exist when using models as predictive tools in DF outbreaks. One of the main limitations for such models as mentioned is the geographic restriction due to data sources, often meteorological stations which might affect the availability of data as well as the spatial applicability. In order to be less constraint on such static datasets, Fuller et al., 2009 [55], included vegetation indices data, as well as sea surface temperatures in relation to El Niño Southern Oscillation (ENSO). Using this model, the authors suggested that a DF outbreak could be predicted with a 40 week advance in Costa Rica, although as discussed by the authors, could be improved with the incorporation of data based on vector population dynamic models as well as seasonal autoregressive modelling methods. Secondly, the differences in input parameters vary due to both natural and artificial factors. Biologically, differences in egg survival time, extrinsic incubation periods, median of lag phase are all directly or indirectly affected by external factors including temperature, humidity or even the immune system on an individual level [15]. The effects of socio-environmental factors on mosquito vectors and transmission of DF are often not immediate, which involves a lag time between exposure to a risk factor and the development of the disease. The inclusion of temporal data allows for the identification of lag times and patterns of transmission over time [15]. Spatial data alone cannot provide the analysis of the temporal kinetics of an outbreak whereas the use of temporal data does not allow for the identification of high risk areas [15]. Ideally, studies should include both spatial and temporal aspects in the analysis to maximise the ability of the resultant equation to predict future outbreaks.

Through the literature review, several interesting parameters were indentified which affect the predictive ability of models, such as the range of transmission of DF being at temperatures of 15–33.2°C, with females feeding more frequently when temperatures are higher [4], [56]. Directly affecting the biology of the vector, temperature also plays an important role on pathogen replication, maturation and period of infectivity. Transmission was also higher in areas where two or more serotypes were found to circulate simultaneously [7]. On the clinical level, facts such as the range in viraemic phase of DF which lasts from 2 to 12 days [57] will affect the precision of the model output. On an artificial level, models can vary due to the choice of regression analysis, the choice of map in terms of digital charting such as raster or vector outputs, although current methods favour vector maps due to their more flexible nature. Within these also lie the choice of geometric factors such as point or polygonal data which in turn will affect the predictive power of the models [58].

Although modelling studies promote the need for a DF vaccine [59], a suitable chimeric vaccine that accounts for all four serotypes of DF is yet to be developed, hence the most effective means of controlling DF is through prevention via vector control. However, many vector control programs deteriorate as the economic condition of most high risk countries is unfavourable [60]. The identification of areas most at risk of DF transmission is essential to ensure the most efficient and effective use of resources for the continuation of vector control and eradication programs. With the predicted socio-environmental changes brought by urbanisation, climate change and globalisation, the regions at risk of transmission along with the economic impact of DF are set to increase. The analysis of previous outbreaks of DF may provide a means of predicting future epidemics in order to establish early warning systems and allocate resources more efficiently [61], [62], [63].

The use of models as a prediction method or part of a surveillance system in terms of early warning have been done for other vector borne diseases such as malaria [64], [65], Rift Valley Fever [24]and Bluetongue virus [66], which after determining the basic transmission pattern in a mathematical model, could then apply climatic events to predict potential outbreaks through Geographical Information Systems (GIS). Such models have been created on a local scale to predict DF outbreaks based on climatic factors as seen in Brazil [67] which used thermal, hydroclimatic, wind, atmospheric pressure, and humidity data as well as in and Puerto Rico [68] where climatic water budget indicators were used to create an early warning system, with the latter study being able to predict a DF outbreak with a three week warning period.

Few studies have been able to collect the necessary amount of spatio and temporal data as well as epidemiological information to analyse the correlation between all these factors. Bayesian spatio-temporal modelling takes into account the effect of covariates and correlations as well as being able to correct for possible errors arising from median estimates of random effects as seen in Yang et al [69]for schistosomiasis, whereby conditional autoregressive models (CAR) were used in the Bayesian smoothing process. Another study addressing the advantages of this modelling technique is seen for dengue in Brazil [70], but as mentioned, data constraints, in this case the lack of socio-economic and meteorological covariates affect the predictive power of the model. Through the analysis of various dengue models and the ability to include varying levels of qualitative and quantitative data, the CAR method seems to have the most potential for developing a robust climate-based epidemic forecasting model.

The identification of high risk areas and trigger factors such as humidity, precipitation, temperature or even travel related disease could allow for early implementation of such interventions so that DF can be effectively and efficiently controlled and prevented. Through the modelling, eventual intervention strategies have been analysed such as the effect of vaccination and the reduction in the number of susceptible individuals [14], [71].

This review highlights the benefits of combining various epidemiological tools focussing on the ability to incorporate climatic, environmental, epidemiological and socio economic factors to create an early warning system. Some recent nationwide systems have shown encouraging results using these methods, as seen in the Chinese Infectious Disease Automated alert and Response System (CIDARS), which uses a combination of a fixed threshold, spatial and temporal detection methods for real time warning of many infectious diseases on a national scale [72]. Information gathering and sharing platforms [73] as seen in the multi-disease data management system interface are promising tools for infectious disease surveillance.

In conclusion, interventions based on early warning systems aimed at preventing DF transmission require significant financial resources and human input, thus it is desirable to target areas and populations at high risk of DF. Modelling processes have shown their potential in identifying such high risk areas. The authors encourage the collection of information on both a spatial and temporal level, along with climatic and socio-environmental variables during future outbreaks of DF, as this will allow for the development of models with maximum predictive capabilities. Multiple and logistic regression models are most often used for analyses, yet as mentioned they are limited due to their inability at accounting for possible confounding factors, auto-correlations, trends and lags in a sufficient manner thus limiting their predictive performance. Recently, the use of spatial and temporal data has enhanced the ability of models to predict outbreaks of DF by allowing for the spatial identification of high risk areas whilst taking into account the temporal kinetics of DF transmission [15].

Certain factors will have to be taken into consideration when modelling DF in light of climate change and travel trends as well as vector habitat alterations. Due to the emerging spread of *Aedes albopictus* [74], models will have to be able to accommodate for the slightly different biology of these mosquitoes, as seen in the spatial modelling using socio environmental indicators in Brazil which had different breteau indices for both *Ae. aegypti* and *Ae. albopictus* [75]. Similarly to other vector borne disease, models vary in their complexity, methodology and area of study which can be very specific and not easily applied to other geographical areas, hence the comparison of less traditional mathematical techniques is more problematic. Transparency is a key factor which will allow for the improved accuracy and performance of models, not only for DF but for many other vector borne diseases which have complex transmission cycles.
